# Closed Incision Negative Pressure Wound Therapy After Resection of Large, Radiated, Soft Tissue Sarcomas

**DOI:** 10.7759/cureus.8055

**Published:** 2020-05-11

**Authors:** Timothy J Irwin, Dennis Orgill

**Affiliations:** 1 Plastic Surgery, Brigham and Women's Hospital/Harvard Medical School, Boston, USA; 2 Plastic Surgery, Brigham and Women’s Hospital, Boston, USA

**Keywords:** negative pressure wound therapy, closed incision, vac, surgical site infections, wounds, radiation, sarcoma soft tissue

## Abstract

Negative pressure wound therapy (NPWT) has revolutionized wound care. Negative pressure therapy (NPT) is now being applied to closed incisions. Closed-incision NPT (ciNPT) management systems apply negative pressure to the incision and structurally stabilize the surrounding tissues. They are thought to be helpful in high-risk surgical closure. Patients with large sarcomas that have been previously radiated are considered to be among the highest risk for postoperative wound complications. We share our experience with ciNPT in two patients after resection of large, previously irradiated invasive sarcomas. In both cases, healing was uncomplicated. ciNPT shows promise of effective and favorable wound healing in early case reports. Additional prospective randomized clinical trials or registry studies will be necessary to provide higher levels of evidence for this technique.

## Introduction

Negative wound pressure therapy (NWPT) has revolutionized the management of complex open wounds [1-2]. The closed- incision negative pressure therapy (ciNPT) system has been developed in which a porous interface is placed over a closed incision in lieu of a traditional surgical dressing (gauze, tape) [3]. It helps to hold incision edges together, remove fluid/exudate, and prevent external contamination. Preclinical studies have reported reduced scar thickness and narrower scar width, increased collagen at the incision site, increased mechanical properties, and increased tensile strength in the ciNPT groups when compared with a traditional surgical dressing [4-5]. In addition, ciNPT was postulated to aid in more favorable wound healing, decreased wound dehiscence, and decreased surgical site infections (SSI) [3,6].

There has been a documented decrease in SSI with ciNPT as compared to traditional surgical dressings across multiple fields: cardiac surgery, obstetrics, plastic surgery, vascular surgery, and orthopedic surgery [7-11]. However, Cochrane systematic reviews have shown low to very low certainty for all outcomes studied related to ciNPT, citing a serious risk of bias and imprecision [12-13]. Conversely, a recent meta-analysis of ciNPT did exhibit a reduction in SSI and identified high-risk patient variables [14]. Based on these data, international multi-disciplinary consensus recommendations were published regarding ciNPT best-use practices [15]. Hence, there is little convincing data and a significant need for higher-level data with a focus on the most at risk wounds based on local factors such as anatomic location, size, and focused wound site exposure to pre-operative radiation.

At our institution, we have treated complex sarcoma cases requiring preoperative radiation therapy +/- neoadjuvant chemotherapy, radical resection, intricate reconstruction, and careful postoperative care. These patients are known to be at high risk (33%-35%) of major wound complications (MWCs) [16-17]. Here, we present two cases where we outline our experience with ciNPT in patients with extremely high-risk primary surgical closures.

## Case presentation

Case one: medial thigh closure after resection of radiation-associated leiomyosarcoma with neoadjuvant radiation/chemotherapy

**Figure 1:**
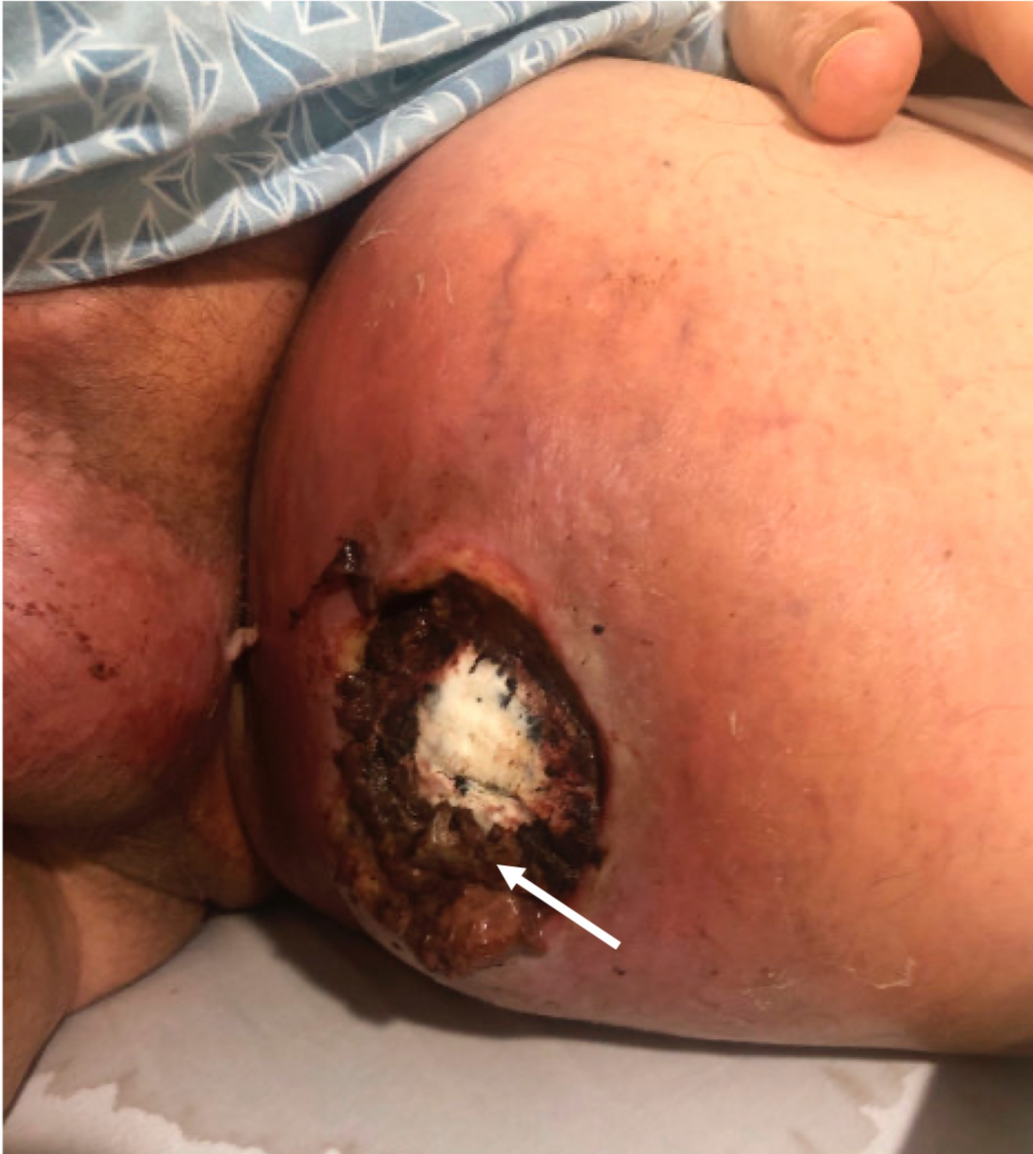
Medial left thigh mass with ulceration and exposure of the necrotic center (white arrow) Notice the erythema and thinning of soft tissues adjacent to the mass from radiation exposure.

**Figure 2:**
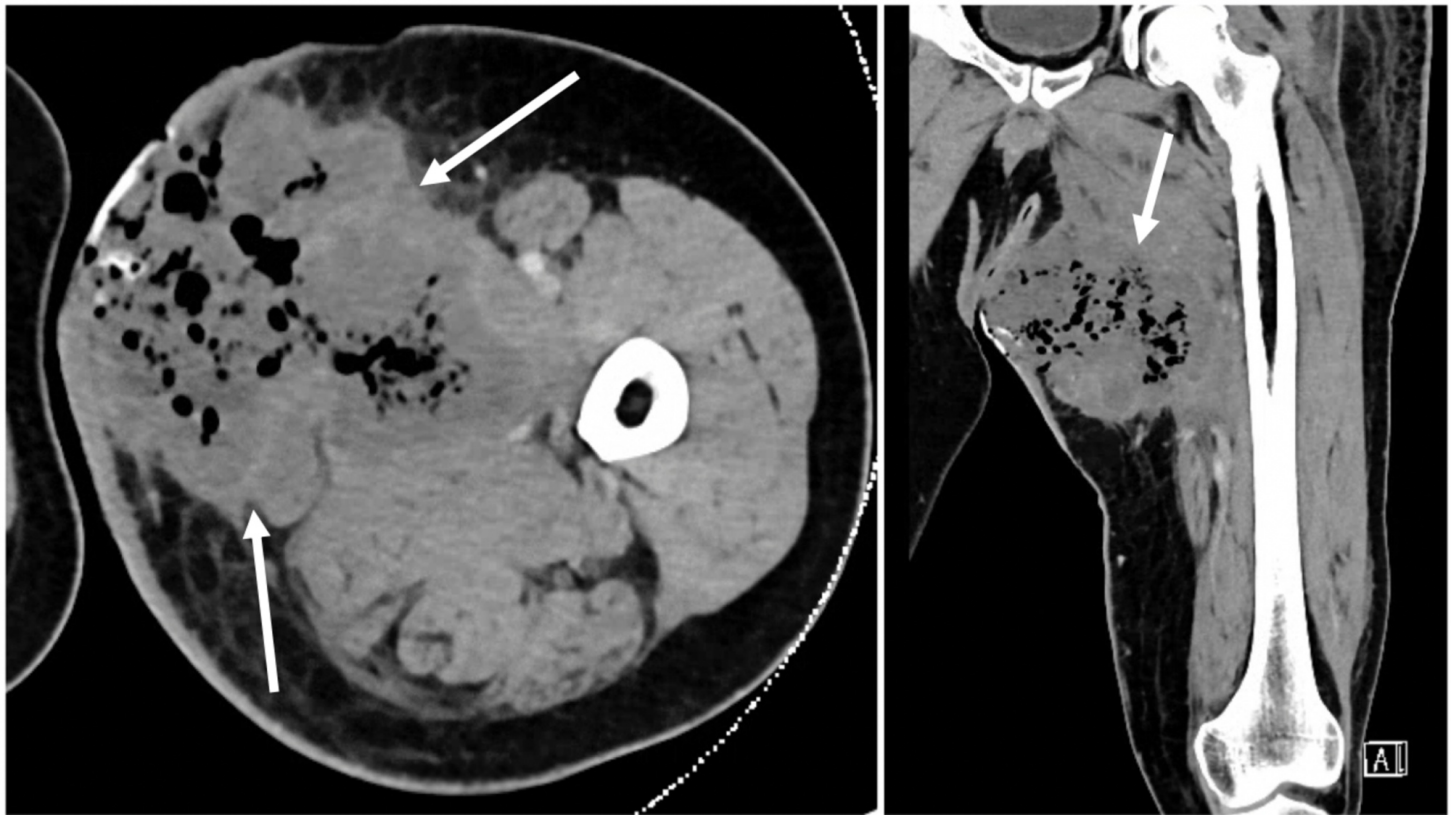
(Left to right: axial view, coronal view) CT scan showing 8.6 x 8.4 x 11.4 cm large, medial proximal thigh soft tissue mass with intralesional gas due to tissue necrosis (white arrows) CT: computed tomography

A 50-year-old male with a history of acute myelogenous leukemia who received total body irradiation (14 Gy) with an allo-stem cell transplant 19 years prior, presented with a fast-growing left groin/medial thigh mass over five months (Figure 1). He was on chronic steroid therapy for graft versus host disease. A magnetic resonance imaging (MRI) demonstrated an 8.6 x 8.4 x 11.4 cm large soft tissue mass. Computed tomography (CT)-guided needle biopsy showed a necrotic high-grade malignant spindle cell neoplasm consistent with radiation-associated leiomyosarcoma (Figure 2). Preoperative radiation, 50 Gy in 25 fractions, was completed two months prior to resection. Preoperatively, he had developed multiple wound infections and a necrotic, open wound with episodic bleeding. 

He underwent radical resection with negative margins, which included partial resection of adductor muscles and patch repair of the superficial femoral artery. A sartorius muscle flap was mobilized to protect the vascular repair. Despite the large area of excised skin, the wound was closable over drains with local tissue rearrangement (length of 30 cm) because of the large volume of subcutaneous tissue resected. A customizable, closed incision NPT dressing (PREVENA^TM^ Incision Management System; KCI, an Acelity Company, San Antonio, TX) was placed over the incision. The patient was admitted postoperatively and underwent seven days of bedrest and continuous ciNPT with 125 mmHg. On postoperative day (POD) 7, the dressing was removed and the patient was discharged to home.

**Figure 3:**
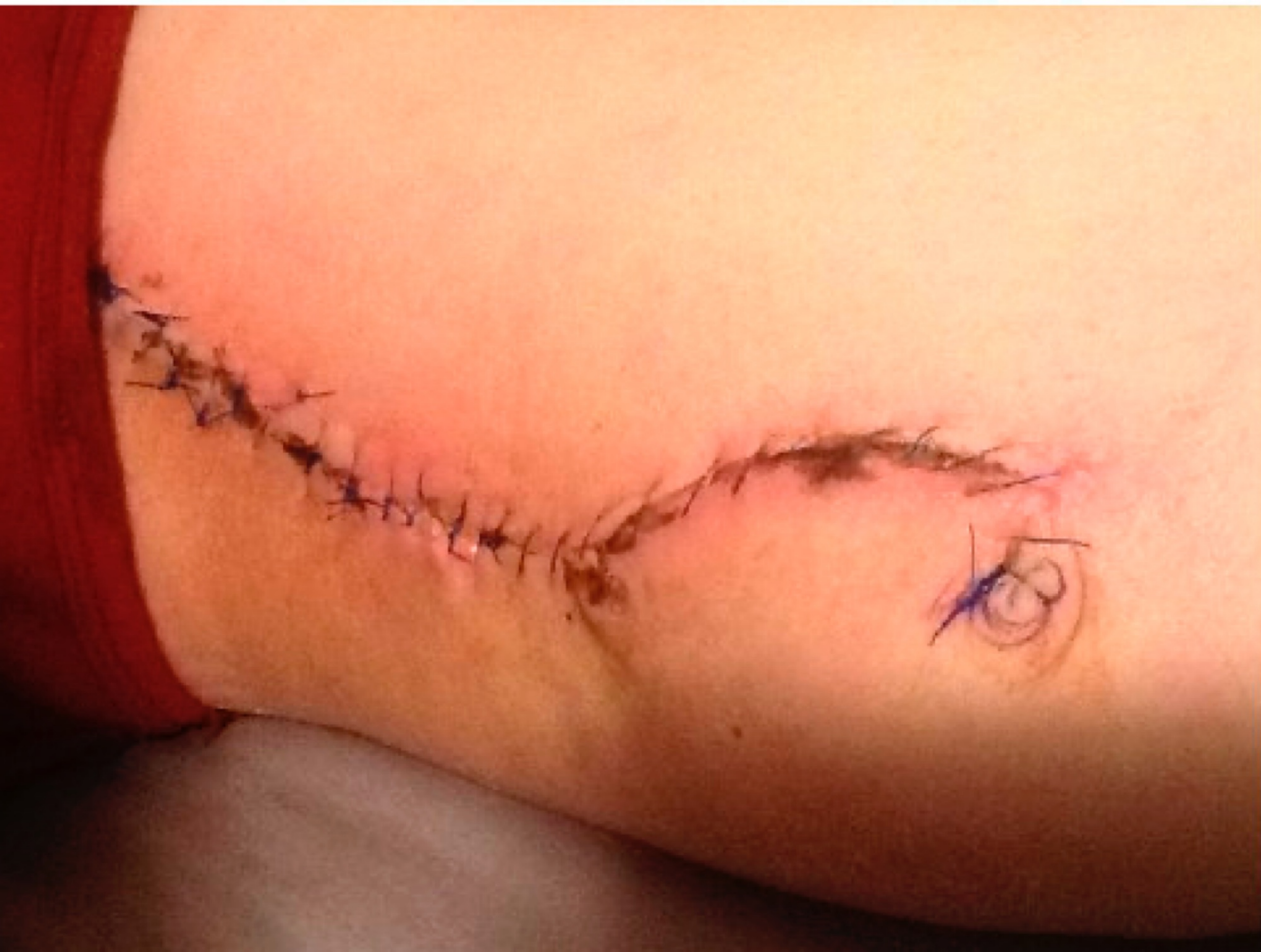
Appearance of surgical incision on medial thigh one month postoperatively, without dehiscence, surgical site infection, or clinically appreciable seroma/hematoma (left side proximal thigh, right side distal thigh)

The patient’s postoperative course has been unremarkable. As of the one-month follow-up, some sutures remain and the incision appears well-healed without dehiscence, SSI, or other complications (Figure 3).

Case two: complex lateral chest wall closure after resection of large radiation-associated osteosarcoma with mesh and latissimus dorsi flap

**Figure 4:**
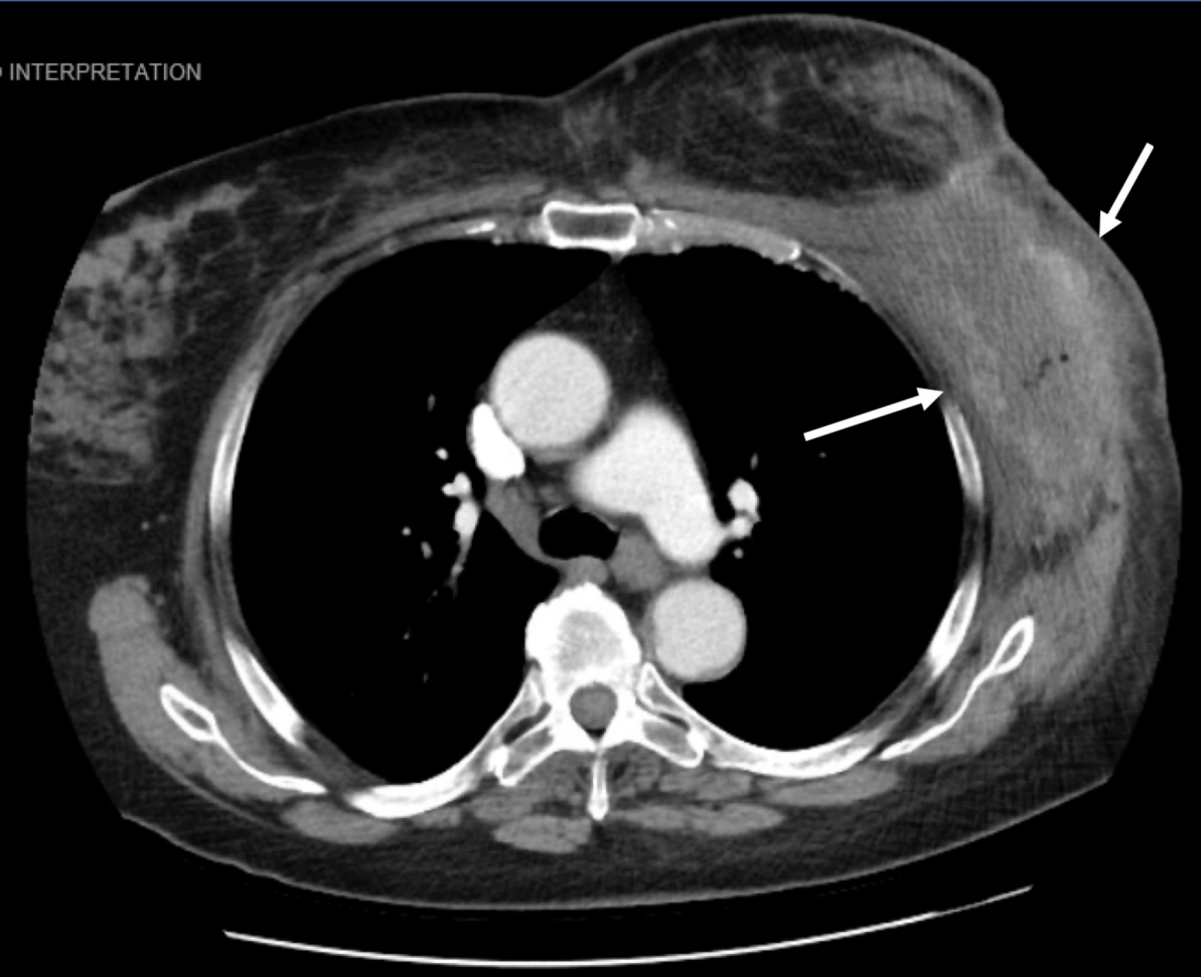
Axial CT scan showing a large, predominantly necrotic mass (white arrows) centered in the left lateral chest wall measuring approximately 9.8 x 5.6 cm CT: computed tomography

**Figure 5:**
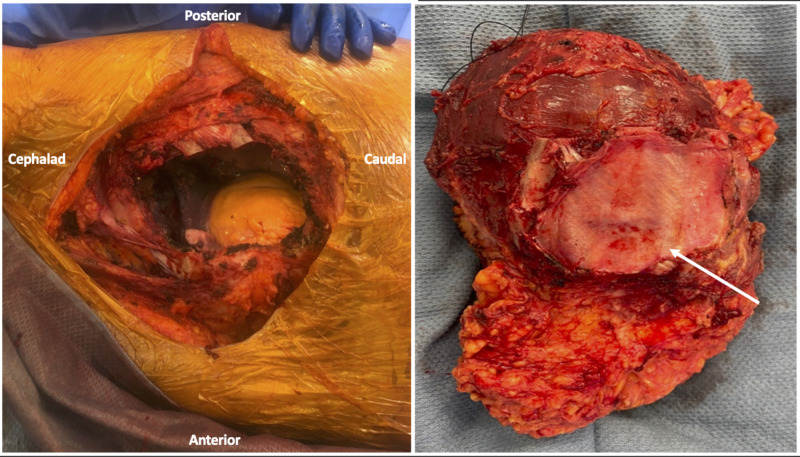
(Left) Intraoperative photo of defect status post radical resection of osteosarcoma en bloc with ribs 3-6, serratus anterior, and breast. (Right) Photo of the deep aspect of the en bloc specimen; ribs and intercostal muscles denoted by white arrow

**Figure 6:**
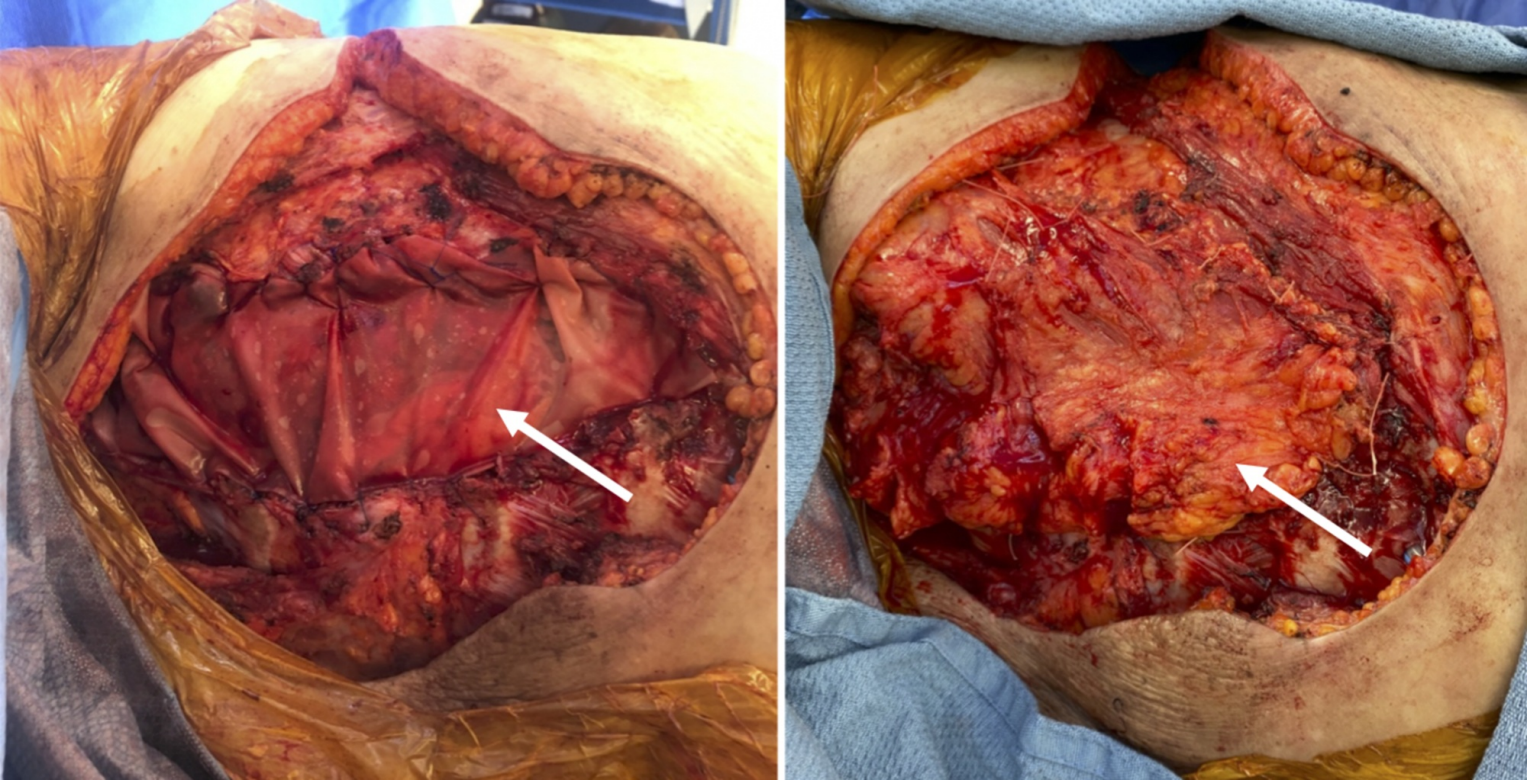
Intraoperative photos Polygatin-910 mesh repair (white arrow) of left-sided chest wall defect (left). Inset of pedicled Latissimus Dorsi flap (white arrow) over polygatin-910 mesh repair (right)

A 69-year-old female with a history of left breast cancer, treated with lumpectomy and radiation therapy (64 Gy) 13 years prior, presented with left breast pain and a palpable mass. Imaging showed a large, necrotic, 10.6 x 4.1 x 5.4 cm mass involving the axillary tails of the pectoralis major and minor muscles with an extension between the latissimus, serratus, and several intercostal spaces (Figure 4). She was treated with neoadjuvant chemotherapy for a likely radiation-induced, high-grade pleomorphic sarcoma with osteosarcomatous differentiation. She developed an open wound and ongoing drainage from the chest wall mass. A radical en bloc* *resection included the left breast, the lateral portion of the pectoralis major muscle, 6 cm portions of ribs 3-6, and the anterior portion of the serratus anterior, leaving a 20 x 15 cm open area of the left lateral chest wall and a 10 x 6 cm defect of the chest wall (Figure 5). A polygatin-910 mesh covered by a pedicled latissimus muscle was used to reconstruct the chest wall and rib cage (Figure 6). Using progressive tension sutures, the skin was mobilized in order to complete closure with a 45 cm “T-shaped” incision, which was then covered with a customizable, closed incision NPWT dressing (PREVENA). The patient was admitted postoperatively and underwent seven days of limited upper extremity abduction and continuous ciNPWT with 125 mmHg. On POD 7, the dressing was removed and the patient was discharged to home.

The patient’s postoperative course has been unremarkable. As of the one-month follow-up, most sutures have been removed and the incision appears well-healed without dehiscence, SSI, or other complications. Radiation oncology plans for postoperative radiation due to positive posterior margins on permanent pathology.

Technical pearls

Application of ciNPT

For large, non-linear incisions, we use the customizable, closed incision NPWT dressings (PREVENA). This allows the exact tailoring of the composite sponge/adhesive dressing to our incision. We center the incision underneath the composite unit and allow approximately 1-2 cm of extension beyond the end of the incision, to ensure the entire incision is covered. There are additional thick, tan adhesive components that we cut and place at the end of the tailored dressing to ensure maximal seal over the incision and then apply the transparent, adhesive film over the composite dressing. Any non-contiguous areas (such as a “T point” or a curve) can be successfully covered by two separate pieces of composite dressing; we suggest that there is about 0.5 to 1 cm of overlap of the composite dressing to ensure adequate suction is applied over this area and there is no gap in the sponge. Of note, we have found that any drains or tubes that are placed must exit 3-4 cm away from the device to prevent the disruption of a proper seal. A quarter size hole is then made in the transparent film over the composite dressing and the SensaT.R.A.C^TM^ (KCI, an Acelity Company, San Antonio, TX) pad (or lily pad) is applied in an orientation that will diminish kinking or pulling. The tubing is then connected to the canister and the machine (either a handheld PREVENA machine or a regular vacuum-assisted closure (VAC) machine on the PREVENA settings) is set to the desired suction. 

Leaks

One preventative measure we employ is the use of Mastisol® (Eloquest Healthcare, Ferndale, MI), a liquid adhesive placed on the adjacent skin, which aids in the initial placement of the transparent, adhesive film; especially in difficult locations (ie. groin, axilla, etc) or in patients with shiny, poor quality skin. Leaks can be found by inspecting and listening to the dressing while it is hooked to suction. Once the leak is identified, it may be covered by additional adhesive film.

## Discussion

The evolution of wound care in the time of NPWT has been rapid. Originally used for clean, complex wounds, the use of NPWT has expanded over the past 20 years to include use over closed incisions after primary closure.

Data exist showing a decrease in SSI with ciNPT as compared to traditional surgical dressings in individual studies [5-11,14]. Based on these data, international multi-disciplinary consensus recommendations were published regarding ciNPT best-use practices [15]. However, Cochrane systematic reviews show there is little convincing data and have shed light on the need for higher-level data with a focus on the most at-risk wounds [12-13].

It has been published that patients with preoperative radiation or previously irradiated wound beds have a significantly increased risk of complications after reconstructive surgery (~33%-35%) [16]. Specifically, in sarcoma patients, further analysis showed that independent risk factors for major wound complications (MWCs) included tumors >10 cm, tumors <3 mm from the skin surface, and vascularized flap/split-thickness skin graft (STSG) closure [17]. Both of our patients possessed all three factors, making their wounds extremely high risk for MWC.

In this patient population, with local pre-operative radiation therapy, there is extremely limited data regarding ciNPT. The experience of Bedi et al. showed decreased wound complications (defined as reoperation, prolonged wound care, or treatment with antibiotics within six months of resection) in 123 patients with lower extremity sarcoma resections with preoperative radiation therapy and chemotherapy when ciNPT was utilized [18]. However, this study was limited to the proximal lower extremity, and ciNPT was not randomized and placed upon surgeon preference/decision.

In each of the cases that we present, the patients were able to heal complex reconstructions without complication despite advanced disease, previous radiation, and tenuous closures. They each had large, irradiated, erosive soft tissue sarcomas and underwent resections requiring extensive reconstructive surgery in notoriously difficult areas to manage wound care (medial thigh/groin and axilla). Each of our cases had three or more independent risk factors for MWCs when comparing them to previously published data. Thus, ciNPT may be a major asset to helping care for this patient population. There is a need for additional comparative data regarding ciNPT, specifically regarding high-risk wounds and focusing on broader clinical outcomes, patient-reported outcomes, and cost-effectiveness.

Of note, we did not experience any complications from the ciNPT systems themselves. ciNPT is considered extremely low risk with only published complications of skin blister formation [19].

## Conclusions

Patients with large, irradiated, soft tissue sarcomas are at a high risk of major wound complications after resection and complex reconstruction. Closed incision NPT shows the potential for improving clinical outcomes in these extremely high-risk, irradiated wounds. Additional prospective randomized clinical trials or registry studies will be necessary to provide further evidence for this technique.
